# The medical student of the future: redefining competencies in a transformative era

**DOI:** 10.3389/fmed.2025.1593685

**Published:** 2025-06-16

**Authors:** João P. Miguez-Pinto, Beatriz Garcia-Rosa, Mateus Maggitti-Bezerril, Clara Ramalho, Stefania L. Garcia, Hugo N. Pustilnik, Daniel Boczar, Katia M. Avena, Bruno B. Andrade

**Affiliations:** ^1^Bahiana School of Medicine and Public Health, Salvador, Brazil; ^2^Oswaldo Cruz Foundation (Fiocruz), Rio de Janeiro, Brazil; ^3^Multinational Organization Network Sponsoring Translational and Epidemiological Research (MONSTER), Salvador, Brazil; ^4^Department of Research, School of Medicine, Salvador University, Salvador, Brazil; ^5^Eastern Virginia Medical School, Norfolk, VA, United States; ^6^Institute of Clinical and Translational Research, Zarns Medical College, Clariens Education, Salvador, Brazil

**Keywords:** artificial intelligence, healthcare, education, technology, innovation

## Abstract

Medical education is undergoing a profound transformation, driven by rapid technological advances, evolving pedagogical strategies, and the shifting demands of healthcare systems globally. This article explores the historical evolution of medical student competencies, evaluates the current state of medical education, and envisions the future competencies required for medical school entrants and graduates. We propose a forward-looking framework for reimagining the medical student of the future, emphasizing the integration of advanced technological literacy, humanistic values, and a global perspective. By redefining these competencies, medical schools can ensure the development of adaptable, empathetic, and innovation-driven physicians for a dynamic healthcare landscape.

## Introduction

The role of medical students has evolved in tandem with significant changes in healthcare delivery, medical knowledge, and educational philosophies. Historically, the medical education system was designed to prepare physicians to treat diseases through a disease-centered approach. Early in the 19th and 20th centuries, students primarily memorized extensive lists of symptoms, treatments, and diseases, reflecting the medical model of the time (1, 2). This model prioritized mastery of a vast body of knowledge over fostering collaborative, patient-centered care. The curriculum was rigid, often promoting individual study, with little emphasis on communication skills or emotional intelligence.

By the mid-20th century, medical education began shifting toward more interactive, evidence-based learning, driven by advances in science, technology, and a greater understanding of the psychological, social, and cultural factors that influence patient care (1). The introduction of problem-based learning (PBL) in the 1960s was a pivotal moment, encouraging medical students to engage more actively with real-world clinical problems and collaborate with peers from various disciplines (3, 4). Concurrently, medical technologies, including imaging, diagnostics, and more recently, digital tools, reshaped the ways physicians diagnose and treat patients, indicating the inadequacy of traditional educational models for preparing students for modern healthcare.

As the 21st century progresses, the healthcare landscape is evolving rapidly, presenting both challenges and opportunities. Advancements such as artificial intelligence (AI), machine learning, precision medicine, telemedicine, and genomics are revolutionizing healthcare delivery and, consequently, medical education (5). Furthermore, the increasing recognition of social determinants of health such as socioeconomic status, education, and living conditions necessitates a broader, more holistic approach to patient care.

The future of medicine will demand that medical students possess competencies in communication, cultural sensitivity, and ethical reasoning. With the growing diversity of patient populations and the global nature of healthcare, future physicians must be equipped to care for patients from various cultural, ethnic, and socioeconomic backgrounds. The emphasis on patient-centered care, which views patients as whole individuals rather than merely a collection of symptoms, will require physicians to develop emotional intelligence and empathy. As such, is it imperative to highlight that the evolving techniques and the active broadening of perspective is crucial to the betterment of medicine and thus, the creation of even greater physicians.

Thus, the medical student of the future must be adaptable, capable of working in multidisciplinary teams, proficient in emerging technologies, and prepared to navigate the complexities of global health. The evolving demands of healthcare systems driven by an aging population, rising chronic diseases, and novel healthcare delivery models mean that medical education must equip students to meet these challenges. An apocryphal anecdote often attributed to Voltaire states that “doctors are men who prescribe medicines of which they know little, to treat diseases of which they know even less, in human beings of whom they know nothing” (6). Though likely more rhetorical than factual, this remark highlights the essential role of medical schools in preparing future physicians through an inclusive, adaptable, and forward-thinking approach to education.

In this article, we examine how competencies required of medical students have evolved and propose a vision for the future. By integrating technological advances, emphasizing patient-centered and holistic care, and addressing the needs of a globalized healthcare system, we offer a comprehensive framework for the medical student of the future. This framework aims to prepare students not only to excel in technical proficiency but also to lead in healthcare innovation, global health, and compassionate patient care.

## The evolution of medical student competencies

Historically, medical education followed a rigid, hierarchical framework that emphasized memorization and standardized knowledge retention over the development of clinical reasoning (2). Students were expected to master extensive factual content through repetitive study and recall-based assessments, reinforcing a model that positioned them as solitary learners within a strictly biological paradigm of disease. This reductionist approach overlooked the psychological, social, and environmental determinants of health, limiting the breadth of medical training (7).

In 1910, the Flexner Report was a pivotal moment in the reform of medical education. It advocated for more scientific and rigorous curricula and set the foundation for global standardization. By emphasizing an evidence-based approach, the report spurred substantial changes in educational policies, elevating the quality standards of medical schools worldwide. This movement toward the integration of scientific evidence in education set the stage for subsequent innovations in medical competencies (8).

A second significant transformation occurred in the 1960s with the introduction of PBL at McMaster University in Canada. This innovative approach challenged passive learning by engaging students in real-world case discussions, fostering peer collaboration, and integrating knowledge across disciplines. PBL established the foundation for competency-based education, transitioning from rote memorization toward problem-solving, adaptability, and critical thinking skills indispensable for contemporary medical practice (9).

As medical knowledge continued to expand in the 1970s and 1980s, medical education began acknowledging the multidimensional nature of health. Unlike traditional curricula, which focused predominantly on diagnosing and treating diseases, a more humanistic approach emerged, redefining the physician’s role. This evolution introduced ethics, communication skills, empathy, and patient-centered care into medical training, reinforcing the necessity of a holistic perspective in clinical practice (4).

In parallel with this humanistic transition, a new challenge surfaced: the exponential expansion of medical knowledge. The sheer volume of emerging research made traditional learning methods increasingly impractical. In 2000, approximately four systematic reviews were published daily; by 2010, this number had risen to 14 per day, and by 2019, over 80 new systematic reviews were published daily (10). This rapid expansion underscored the urgency for students to develop skills in information retrieval, critical appraisal, and evidence-based application rather than relying solely on memorization.

Before medical education could fully integrate competency-based models, AI emerged as a transformative force, reshaping both medical practice and education. Digital health platforms, simulation-based learning, and virtual reality technologies now provide immersive, hands-on experiences, allowing students to refine diagnostic reasoning and procedural skills in controlled, low-risk environments before engaging in real clinical scenarios (11, 12).

Furthermore, the increasing emphasis on cultural competence in medical curricula reflects the necessity of preparing physicians to navigate the diverse social, economic, and cultural determinants of health. As globalization continues to influence healthcare delivery, the ability to practice within multicultural contexts has become an essential competency, ensuring that future physicians are equipped to address the complexities of patient care in a rapidly evolving world (13).

Ultimately, medical education has evolved from a rigid, memorization-based system to an adaptive, evidence-driven framework that prioritizes clinical reasoning, patient-centered care, and technological integration, ensuring that future physicians are well-prepared to meet the demands of modern medicine.

## The future medical student: a vision

The medical student of the future will be shaped by rapid advances in pedagogy, technology, and evolving societal demands. Admission criteria will increasingly emphasize proficiency in data analytics, AI, and digital health platforms, recognizing their pivotal role in integrating AI into clinical practice, enhancing diagnostic accuracy, and improving patient outcomes. Beyond technical expertise, adaptability and strong communication skills will be essential, equipping future physicians to navigate complex healthcare environments while maintaining a patient-centered approach.

Interdisciplinary knowledge will also become a defining feature of future medical school applicants. Backgrounds in bioinformatics, global health, and medical ethics will offer valuable perspectives for addressing intricate healthcare challenges. As medical education evolves, it will prioritize lifelong learning, holistic care, and global awareness, ensuring adaptability to rapid scientific advancements. Continuous professional development will remain integral to maintaining clinical competence, with training focusing on self-directed learning and the seamless integration of emerging technologies into clinical practice.

Technological advancements, particularly in robotics and AI, are rapidly reshaping the medical landscape. While robotic surgery has traditionally been introduced at the graduate medical education level, recent discussions have emphasized the importance of early exposure during undergraduate medical training to better prepare students for surgical residency (14, 15). Meanwhile, AI applications are already a reality in medical education, necessitating an immediate response from academic institutions. A study using the Unified Theory of Acceptance and Use of Technology model, conducted across 12 universities and 33 hospitals, found that 59.65% of undergraduate medical students had prior experience with medical AI (16). Additionally, a multicenter study involving 3,018 medical students revealed that 93.8% of participants supported structured AI training within medical curricula (17). These findings underscore the necessity of integrating robotics and AI training into medical education to equip future physicians with the competencies required for an increasingly technology-driven healthcare environment.

However, despite the potential of AI and robotics in medical education, there are several barriers to their widespread integration. Resource constraints, including the high costs of acquiring and maintaining AI technologies and robotic equipment, pose a significant challenge, especially for institutions in low- and middle-income countries. In addition, there are ethical concerns related to data privacy, the use of AI in decision-making, and the potential dehumanization of medical training (18).

To mitigate ethical concerns, medical schools must implement clear guidelines on the ethical use of AI and robotics, emphasizing the importance of preserving humanistic elements in patient care. Training in AI ethics, data privacy, and informed consent should be integrated into the curriculum to ensure future physicians are equipped to handle the ethical dilemmas that may arise from AI-driven medical decisions. Additionally, ensuring that AI and robotics complement rather than replace human interaction in clinical settings will be critical to maintaining the patient-centered focus of medical education.

Leadership will also play a pivotal role in shaping innovative and sustainable healthcare systems. Historically, medical education has not emphasized managerial and organizational skills; however, this trend is shifting. Over the past decades, leadership competencies have gained recognition as essential attributes in medical training, with institutions worldwide increasingly incorporating leadership development into their curricula (19, 20).

In addition to clinical, technological, and leadership skills, future physicians must be prepared to take an increasingly active role in disease prevention and health promotion. As health systems shift from reactive to preventive models, physicians are expected not only to identify risk factors and implement preventive strategies but also to engage with communities in promoting healthy behaviors (21). This shift requires medical schools to increasingly incorporate and prioritize public health principles and preventive care in undergraduate training, thus preparing professionals capable of integrating biomedical knowledge with population health perspectives.

In summary, the medical student of the future will embody a unique blend of technological proficiency, interdisciplinary knowledge, leadership skills, and adaptability. However, the successful integration of AI and robotics in medical education will depend not only on technological advances but also on overcoming resource limitations and addressing ethical concerns. These attributes will enable them to navigate and shape an increasingly complex and interconnected global healthcare landscape.

## The role of medical schools in shaping the future

Medical schools play a fundamental role in preparing future physicians for a rapidly evolving healthcare landscape marked by technological advancements and increasing globalization. To fulfill this mission, curricula must be restructured to integrate emerging technologies such as AI, robotics, and telemedicine. Incorporating machine learning courses into medical education has been shown to enhance personalized learning experiences and facilitate realistic simulations, ultimately improving educational outcomes (22). Furthermore, these technological skills align with global health initiatives and emphasize the need for digitally literate physicians capable of navigating interconnected health systems. Partnerships between international institutions can help standardize training in emerging technologies, ensuring that skills learned locally have a global impact.

Simulation-based training further enhances real-world preparedness by providing immersive, hands-on experiences that develop clinical competencies in a controlled environment. Additionally, personalized learning approaches are transforming medical education by tailoring instruction to individual student needs. Adaptive learning platforms, leveraging data-driven insights, enable students to progress at their own pace, ensuring mastery of core competencies. These platforms have demonstrated effectiveness in improving knowledge retention and fostering individualized learning experiences. Recognizing the need for curricular innovation, the American Medical Association (AMA) has launched initiatives to support educational reform, focusing on competency-based assessments, flexible learning pathways, and enhanced clinical experiences within healthcare systems (23).

Beyond technological advancements, the expansion of humanities and ethics education remains essential for cultivating empathy, creativity, and ethical reasoning in medical professionals. A balanced integration of technical expertise and humanistic values is crucial for training well-rounded physicians capable of delivering compassionate, patient-centered care in increasingly complex clinical settings.

Global collaboration is becoming a cornerstone of medical education, offering significant benefits for individuals, academic institutions, and society. International partnerships expose students to diverse healthcare systems, broadening their perspectives and enhancing cultural competence. These international exchanges directly contribute to the global competencies needed for modern physicians, ensuring they are prepared to practice in various cultural contexts and address global health challenges. These exchanges deepen their understanding of global health challenges, such as infectious diseases, maternal health, and the impact of climate change on healthcare delivery. Programs facilitated by the International Federation of Medical Students’ Associations (IFMSA) emphasize cross-border collaboration, providing students with opportunities for professional exchanges worldwide. Studies indicate that international experiences in healthcare positively influence career choices, increasing the likelihood of pursuing primary care and fostering a commitment to serving vulnerable communities (24, 25). Such experiences are critical in preparing future physicians to address global health issues and function effectively in diverse cultural settings, particularly as healthcare systems become increasingly interconnected.

In conclusion, medical schools must proactively adapt to the evolving healthcare landscape by integrating technological advancements, embracing personalized learning approaches, and fostering global collaboration. Through these efforts, they will equip future physicians with the essential skills, knowledge, and cultural competence necessary to deliver compassionate, patient-centered care while addressing the complexities of a rapidly transforming global healthcare environment.

## Conclusion

The future of medical education demands a fundamental shift in the competencies required of physicians to address the evolving challenges of a dynamic, global healthcare system. Future physicians will need to combine technical expertise with emerging technologies, such as artificial intelligence, while maintaining a patient-centered, humanistic approach. Equally important, they must increasingly act proactively as health promoters and prevention agents, broadening the scope of expected competencies and aligning with global trends in public health and preventive medicine.

A global perspective, interdisciplinary collaboration, and cultural competence will also be essential as healthcare becomes more interconnected and diverse. Medical schools must adapt their curricula to prepare students for new health challenges, scientific advancements, and patient expectations. By integrating technology, fostering continuous learning, and developing ethical leadership, medical schools can equip future physicians to innovate, lead, and provide compassionate care. Ultimately, the medical student of the future will not only diagnose and treat but will also drive healthcare transformation in a complex, rapidly changing world.
